# Contributions of tropodithietic acid and biofilm formation to the probiotic activity of *Phaeobacter inhibens*

**DOI:** 10.1186/s12866-015-0617-z

**Published:** 2016-01-05

**Authors:** Wenjing Zhao, Christine Dao, Murni Karim, Marta Gomez-Chiarri, David Rowley, David R. Nelson

**Affiliations:** Department of Cell and Molecular Biology, University of Rhode Island, 120 Flagg Rd., Kingston, RI 02881 USA; Biomedical and Pharmaceutical Sciences, University of Rhode Island, Kingston, RI 02881 USA; Fisheries, Animal and Veterinary Sciences, University of Rhode Island, Kingston, RI 02881 USA; Present Address: Department of Microbiology and Immunology, Harvard Medical School, Boston, MA 02115 USA; Present Address: Department of Chemistry and Biochemistry, University of Massachusetts Dartmouth, Darmouth, MA 02747 USA; Present Address: Department of Aquaculture, Faculty of Agriculture, Universiti Putra Malaysia, 43400 Serdang, Selangor Malaysia

**Keywords:** *Phaeobacter inhibens*, Tropodithietic acid, Biofilm formation, Probiotic, Marine pathogens, *Vibrio tubiashii*, *Vibrio anguillarum*, Oyster disease, ClpX, ExoP

## Abstract

**Background:**

The probiotic bacterium *Phaeobacter inhibens* strain S4Sm, isolated from the inner shell surface of a healthy oyster, secretes the antibiotic tropodithietic acid (TDA), is an excellent biofilm former, and increases oyster larvae survival when challenged with bacterial pathogens. In this study, we investigated the specific roles of TDA secretion and biofilm formation in the probiotic activity of S4Sm.

**Results:**

Mutations in *clpX* (ATP-dependent ATPase) and *exoP* (an exopolysaccharide biosynthesis gene) were created by insertional mutagenesis using homologous recombination. Mutation of *clpX* resulted in the loss of TDA production, no decline in biofilm formation, and loss of the ability to inhibit the growth of *Vibrio tubiashii* and *Vibrio anguillarum* in co-colonization experiments. Mutation of *exoP* resulted in a ~60 % decline in biofilm formation, no decline in TDA production, and delayed inhibitory activity towards *Vibrio* pathogens in co-colonization experiments. Both *clpX* and *exoP* mutants exhibited reduced ability to protect oyster larvae from death when challenged by *Vibrio tubiashii*. Complementation of the *clpX* and *exoP* mutations restored the wild type phenotype. We also found that pre-colonization of surfaces by S4Sm was critical for this bacterium to inhibit pathogen colonization and growth.

**Conclusions:**

Our observations demonstrate that probiotic activity by *P. inhibens* S4Sm involves contributions from both biofilm formation and the production of the antibiotic TDA. Further, probiotic activity also requires colonization of surfaces by S4Sm prior to the introduction of the pathogen.

**Electronic supplementary material:**

The online version of this article (doi:10.1186/s12866-015-0617-z) contains supplementary material, which is available to authorized users.

## Background

Infections by pathogenic marine bacteria are a major problem for both the shellfish and finfish aquaculture industries, causing severe disease and high mortality, which seriously affect aquaculture production and cause significant economic loss [1]. This problem particularly affects the survival and growth of fish and shellfish during the larval and juvenile stages [1, 2]. Opportunistic pathogens from the Vibrionaceae and at least one member of the *Roseobacter* clade cause disease in a variety of shellfish [3, 4]. For example, *Vibrio tubiashii*, a reemerging pathogen of larval bivalve mollusks that causes invasive and toxigenic disease, has been responsible for massive mortalities among larval Pacific oysters (*Crassostrea gigas*) in hatcheries on the west coast of the United States [4]. Additionally, *Roseovarius crassostreae*, a member of the *Roseobacter* clade and the causative agent of juvenile or *Roseovarius* oyster disease (JOD or ROD), can cause high mortalities in juvenile eastern oysters (*Crassostrea virginica*) in the northeastern United States during the summer when water temperatures are ≥20 °C [5]. Although antibiotics and vaccines can be used to control some infectious diseases in aquaculture, they have some distinct disadvantages and limitations. Use of antibiotics increases the risk of development and transfer of antibiotic resistance [6]. Vaccines, which rely on an adaptive immune response, are only effective for vertebrate organisms and cannot be used to protect shellfish [7].

Probiotics represent a promising alternative strategy to control infection and some probiotic strains are already used commonly in aquaculture as biological control agents in finfish and shellfish [8, 9]. For example, the probionts *Bacillus subtilis* and *Bacillus licheniformis* are widely used in shrimp aquaculture to provide beneficial effects potentially including improved health and water quality, control of pathogenic bacteria and their virulence, stimulation of the immune system and improved growth [10]. Several *Phaeobacter* species have been shown to be effective probiotics for both finfish and shellfish. For example, D’Alvise et al. [11] demonstrated that *Phaeobacter* can be used as a probiotic treatment to reduce the density of the fish pathogen *Vibrio anguillarum* in cultures of cod larvae, resulting in the reduction of mortality by vibriosis. The probiotic activity was dependent upon the production of tropodithietic acid (TDA) by *P. gallaeciensis*. Further, D’Alvise et al. [12] demonstrated that a different TDA-producing strain of *Phaeobacter* was able to reduce or eliminate *V. anguillarum* from a combined liquid-surface system. These and other studies strongly suggest that antagonistic interactions by probiotic bacteria against marine pathogens may be useful in protecting commercially important species of shellfish and finfish from infectious disease.

*Phaeobacter inhibens* is gram-negative α-*Proteobacteria* from the *Roseobacter* clade. The *Roseobacter* clade, an important member of the marine microbiota, accounts for ~4 % to as much as ~40 % of bacterial DNA from the ocean and plays an important role in the organic sulfur cycle of the ocean [13–15]. Several species in this clade exhibit inhibitory activity against the growth of marine pathogens, including *V. anguillarum*, *V. tubiashii* and *R. crassostreae* [11, 12, 16]. Additionally, several potentially probiotic species from the *Roseobacter* clade can be routinely isolated from larval production facilities for turbot [17]. Further, *Phaeobacter* species are typically excellent biofilm formers, colonizing a variety of surfaces including the walls of rearing tanks, microalgae, the skin of finfish, and the shells of mollusks [12, 18, 19]. Although, biofilm formation is thought to be essential for probiotic activity by a variety of mechanisms including competition for adhesion sites, oxygen, nutrients, and by preventing contact between pathogens and hosts [20], the role of biofilm formation in the probiotic activity of *Phaeobacter* species against shellfish pathogens has not been thoroughly investigated.

Previously, we isolated *P. inhibens* S4 from the inner shell surface of a healthy oyster [16]. This bacterium is a short rod with 1–2 flagella on one or both poles. It has pleiomorphic morphology and will elongate into long rods and filaments under specific conditions (low salt concentration, static incubation, stationary phase). It can form rosettes and is an excellent biofilm former and a dominant colonizer of surfaces in marine environments. *P. inhibens* S4Sm is a spontaneous streptomycin-resistant mutant of the parental S4. When S4Sm was used as a potential probiotic treatment of oyster larvae, it showed strong anti-pathogen activity and increased host survival [16], but the actual mechanisms of probiotic activity used by this isolate are not fully understood.

In this study we examined the roles of biofilm formation and TDA production in probiotic activity of *P. inhibens* S4Sm in oysters challenged by the pathogen, *V. tubiashii*. In order to determine the contributions of TDA production and biofilm formation to the probiotic activity of S4Sm, mutations in *clpX* (which blocks TDA biosynthesis [21]) and an exopolysaccharide biosynthesis gene (*exoP*) (potentially involved in biofilm formation) were created by insertional mutagenesis. The effects of these mutations upon TDA production, biofilm formation and probiotic activity were determined.

## Results

### *P. inhibens* S4Sm secretes the antibiotic tropodithietic acid

**Fig. 1:**
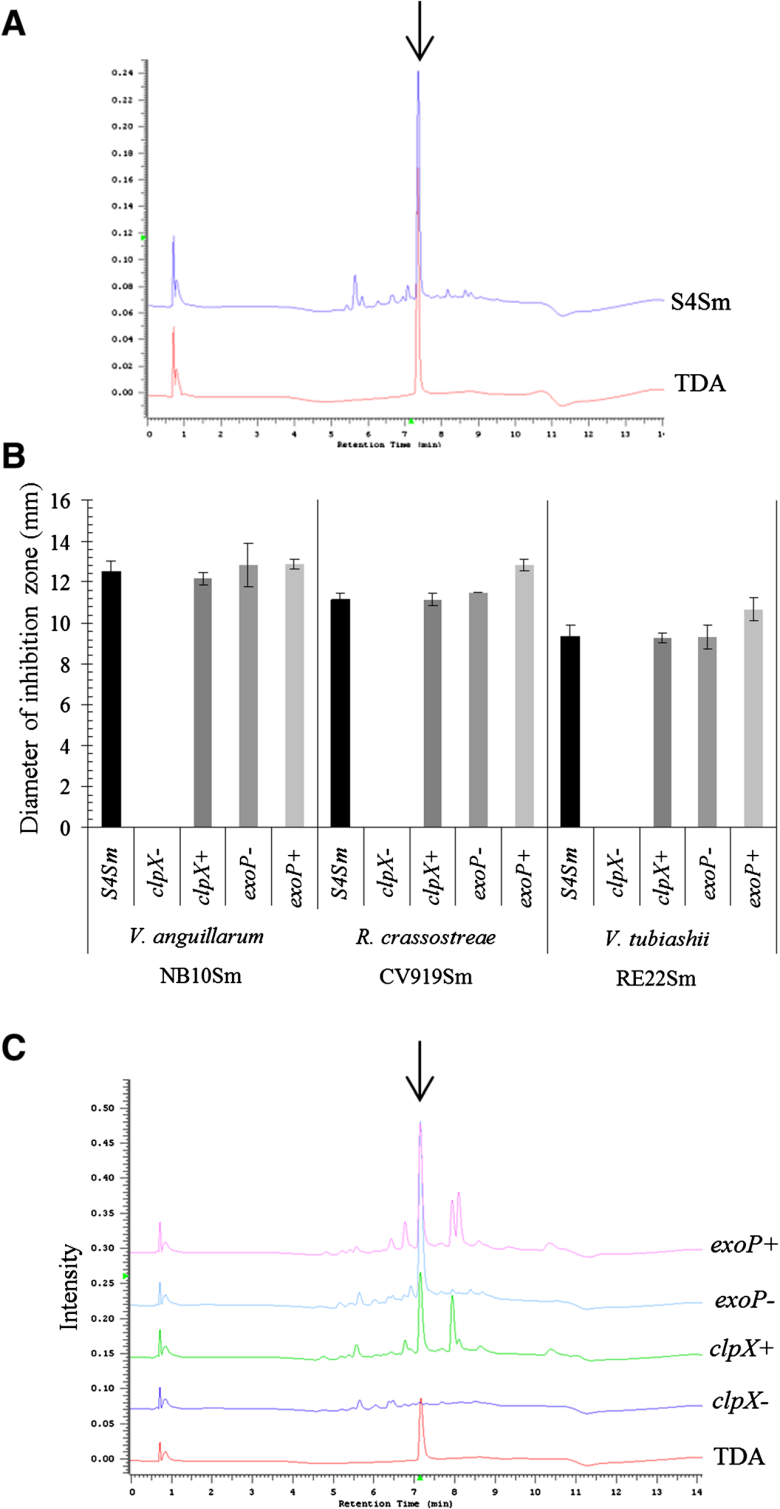
Reversed-phase HPLC chromatograms of ethyl acetate extracts from *Phaeobacter* strains to detect TDA. **a** Authentic TDA and extract from wild type strain S4Sm. **b** Inhibition zone assay of S4Sm, *clpX* mutant (*clpX*-), *clpX* complement (*clpX*+), *exoP* mutant (*exoP*-) or *exoP* complement (*exoP*+) on YP30 plates coated by *V. anguillarum* (NB10Sm), *V. tubiashii *(RE22Sm) or *R. crassostreae* (CV919Sm) after 24 h at 27 °C. **c** Authentic TDA and extracts from *clpX* mutant strain (*clpX*-), *clpX* complement (*clpX*+), *exoP* mutant strain (*exoP*-), exoP complement (*exoP*+). The data presented are averages of two independent experiments and each independent experiment has three replicates. Error bars represent one standard deviation

Bioassay-guided fractionation of *P. inhibens* supernatants resulted in the purification of a single secondary metabolite possessing antimicrobial activity. The molecule was identified as tropodithietic acid (TDA) based upon a molecular ion of [M + H]^+^ = 213 [13] and comparison of ^1^H NMR chemical shift data (500 MHz, C_6_D_6_) with literature values [22] (Additional files 1, 2 and 3). All assays described below were conducted with this purified TDA. UHPLC analysis data (Fig. 1a) confirmed that TDA was present in S4Sm supernatant.

### Differential sensitivities of marine pathogens to TDA

We examined the relative sensitivities of three pathogens of marine organisms, *V. anguillarum* NB10Sm, *V. tubiashii* RE22Sm, and *R. crassostreae* CV919Sm, to *P. inhibens* S4Sm by looking at the inhibition of growth around a colony of S4Sm. *V. anguillarum* NB10Sm was most sensitive to S4Sm with largest zone of inhibition (ZOI) (diameter = 12.5 ± 0.5 mm); *R. crassostreae* exhibited slightly less sensitivity to S4Sm (ZOI =11.2 ± 0.3 mm); and the least sensitive pathogen to S4Sm was *V. tubiashii* RE22Sm (ZOI = 9.2 ± 0.6 mm) (Fig. 1b). These data were consistent with the results for minimum inhibitory concentration (MIC) of TDA against each of the three pathogens: the MIC for TDA against NB10Sm was 1.25 μg/ml, against *R. crassostreae* the MIC was 5 μg/ml, and against RE22Sm the MIC was 6.25 μg/ml.

### Biofilm formation by *P. inhibens* S4Sm

S4Sm formed thick biofilms on glass, as determined using the crystal violet staining assay. The OD_580_ value for the S4Sm biofilm after 60 h was ~4.0 at 27 °C under static conditions (Table 1). In contrast, all three marine pathogens (*V. anguillarum, V. tubiashii,* and *R. crassostreae*) used in this study formed biofilms that were between 13.4-14.9 % of the S4Sm (Table 3) (*P* < 0.05). These data suggested that S4Sm was able to form a thick, dense biofilm matrix on glass coverslips and tubes.

**Table 1:** Quantification of biofilm formation by measuring optical density at 580 nm (OD_580_) of crystal violet dye attached to the cells forming biofilms on glass tubes at 27 °C under static conditions at 60 h

Strains	OD_580_ (±SD)^a^
*P. inhibens* S4Sm	3.89 ± 0.06
*P. inhibens* WZ10 (*clpX*-)	3.90 ± 0.12
*P. inhibens* WZ11 (*clpX*+)	4.0 ± 0.06
*P. inhibens* WZ20 (*exoP*-)	1.60 ± 0.09^b^
*P. inhibens* WZ21 (*exoP*+)	3.90 ± 0.10
*V. anguillarum* NB10Sm	0.58 ± 0.02^b^
*V. tubiashii* RE22Sm	0.54 ± 0.02^b^
*R. crassostreae* CV919Sm	0.52 ± 0.08^b^

^a^Biofilm formation quantified by crystal violet dye assay as described in the Materials and Methods. The data presented are the average of two independent experiments, each with three replicates. SD = standard deviation

^b^Statistically significant difference (P <0.05) compared to S4Sm

### Effect of *clpX* gene mutation on TDA production

In order to examine the roles of TDA production and biofilm formation in the probiotic activity of S4, we constructed mutations in the *tdaA*, *tdaB,* and *tdbD,* genes, previously shown to be part of the TDA biosynthesis pathway [21]. These mutants not only lost TDA production, but also were defective for biofilm formation (Additional file 4). To differentiate the roles of TDA production and biofilm formation in probiotic activity in oysters, we constructed mutants deficient in either TDA synthesis or biofilm production. It was previously shown that mutation in *clpX* resulted in the loss of TDA production in *Phaeobacter* sp. strain 27–4 [21]. The *clpX* gene was PCR amplified and sequenced. The derived amino acid sequence was compared (using BLASTx) to other *clpX* genes already available for *Phaeobacter* strains in the non-redundant protein GenBank database. The S4Sm ClpX protein has 100 % identity to other *P. inhibens* ClpX proteins. The *clpX* gene was found to encode a 408 amino acid ATP-dependent protease ATP-binding subunit and is part of the ClpXP multimer (Accession number: WP_014874379). Mutation of *clpX* by insertional mutagenesis resulted in the loss of TDA production. UHPLC analysis data (Fig. 1c) showed that no TDA was present in *clpX* mutant supernatant. Further, there were no inhibition zones around the *clpX* mutant cells when tested against the three pathogens, *V. anguillarum* NB10Sm, *V. tubiashii* RE22Sm, and *R. crassostreae* CV919Sm (Fig. 1b). Additionally, culture supernatant from the *clpX* mutant was not able to kill NB10Sm cells (Table 2). Complementation of the *clpX* gene restored TDA production (Fig. 1c) and anti-*Vibrio* activity (Fig. 1b and Table 2). Mutation of *clpX* did not result in defective biofilm formation (Table 1). The *clpX* mutant and the *clpX* complement exhibited the same growth rate and final cell density as the wild type when grown in YP30 under shaking and static conditions (Additional file 5).

**Table 2:** Killing ability of culture supernatant of various *P. inhibens* strains against *V. anguillarum* NB10Sm cells^a^

Treatment	Surviving *V. anguillarum* cell density (CFU/ml) after the treatment (±SD^b^)
NSS^c^ (negative control)	40.7 (±3.8) × 10^7^
S4Sm culture supernatant	<10
WZ10 (*clpX-*) culture supernatant	41.3 (±1.5) × 10^7^
WZ11 (*clpX+*) culture supernatant	<10
WZ10 (*clpX-*) culture supernatant plus TDA	<10
WZ20 (*exoP-*) culture supernatant	<10
WZ21 (*exoP*+) culture supernatant	<10

^a^Culture supernatant from each strain collected after two-day incubation. The data presented are from a representative experiment of two independent experiments

^b^SD = standard deviation

^c^NSS: Nine salts solution

### Effect of *exoP* gene mutation on biofilm formation

In order to develop a strain of S4 defective in biofilm formation but able to produce TDA, the *exoP* gene, which encodes an exopolysaccharide biosynthesis domain protein (based on Tigrfam classification systems), was identified in *P. inhibens* S4Sm strain. Mutation of *exoP* resulted in decreased biofilm formation, with the *exoP* mutant exhibiting only ~40 % of the wild type level of biofilm formation (Table 1) (P < 0.05). Complementation of *exoP* gene restored biofilm formation to wild type level (Table 1). Mutation of *exoP* did not result in defective TDA production (Fig. 1b). The *exoP* mutant and the *exoP* complement exhibited the same growth rate and final cell density as the wild type when grown in YP30 under shaking and static conditions (Additional file 5).

### Effect of *clpX* and *exoP* mutations on the ability of *P. inhibens* biofilms to antagonize colonization of coverslips by *Vibrio* species

**Fig. 2:**
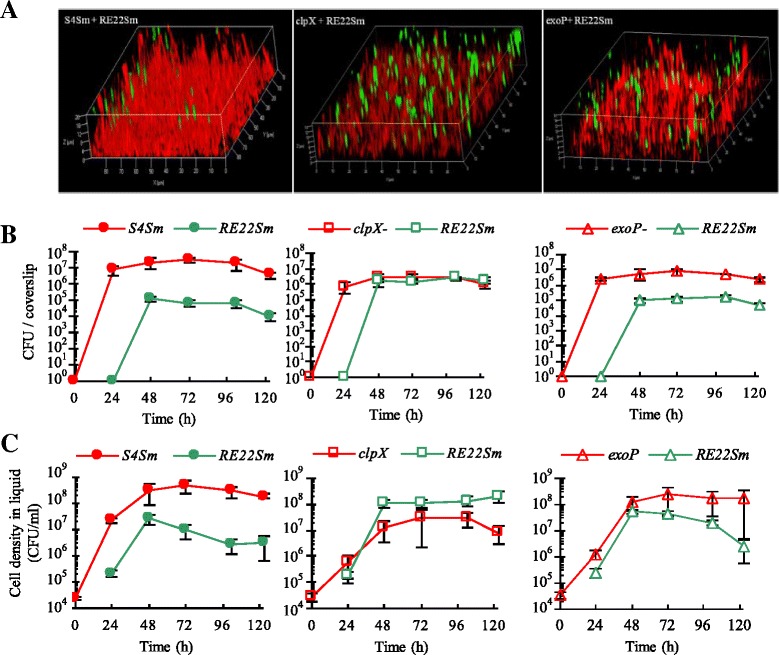
Competition assay between OFP-labeled *P. inhibens* S4 strains and GFP-labeled *V. tubiashii. P. inhibens* stains were allowed to grow and form biofilms on the glass surfaces for 24 h before the addition of *V. tubiashii* RE22Sm-GFP. The mixed cultures are S4Sm-OFP with RE22Sm-GFP, *clpX*-OFP with RE22Sm-GFP and *exoP*-OFP with RE22Sm-GFP. **a** Merged confocal microscopy images of mixed biofilm development by OFP-producing strains (S4Sm, *clpX* mutant *and exoP* mutant) and GFP-producing *V. tubiashii* (RE22Sm) strain on the surface of glass coverslip at 72 h. The data presented are from a representative experiment of two independent experiments. **b** Growth of sessile *P. inhibens* S4Sm strains (S4Sm, clpX, and exoP) and *V. tubiashii* RE22Sm in a co-culture system. **c** Growth of planktonic *P. inhibens* S4Sm strains (S4Sm, clpX, and exoP) and *V. tubiashii* RE22Sm in a co-culture system. The data presented are average of two independent experiments and each independent experiment has three replicates. Error bars represent one standard deviation

The *clpX* mutant (no TDA production and normal biofilm) and the *exoP* mutant (normal TDA production and reduced biofilm) allowed us to examine the relative roles of biofilm formation and TDA production on the ability of 24 h biofilms of S4Sm to: 1) antagonize colonization of glass surfaces by *V. tubiashii* RE22Sm and 2) decrease the cell density (CFU/ml) of the pathogen in the liquid culture media. When a co-colonized glass coverslip was examined after 72 h of incubation by laser scanning confocal microscopy, more RE22Sm cell clusters were observed in the biofilm matrix of the *clpX* mutant than in the biofilm matrix of either S4Sm wild type or *exoP* (Fig. 2a). These observations were reflected in the viable cell counts of the *V. tubiashii* RE22Sm in both biofilms (sessile cells; Fig. 2b) and in suspension (planktonic cells; Fig. 2c) when grown in the presence of biofilms of *P. inhibens* S4Sm wild type, the *clpX* mutant or the *exoP* mutant. For example, as shown in Fig. 2b at 123 h, the number of viable RE22Sm in the biofilm on a coverslip was 1 × 10^4^ CFU when precolonized with S4Sm. In contrast, the number of RE22Sm cells increased 180-fold (to 1.8 × 10^6^ CFU/coverslip) when grown in the presence of the *clpX* mutant. This was about the same number of cells on a coverslip as when RE22Sm was allowed to colonize alone (2.0 × 10^6^ CFU/coverslip). Further, when grown in the presence of the *exoP* mutant the number of viable RE22Sm cells on the coverslip was 4.5-fold higher (4.5 × 10^4^ CFU/coverslip) than in the presence of S4Sm cells (1 × 10^4^ CFU/coverslip) (Fig. 2b). This is a significant difference in RE22Sm biofilm formation (P < 0.05). In suspension, the cell density of RE22Sm reached 2 × 10^8^ CFU/ml under conditions of precolonization by the *clpX* mutant; this was similar to the density of RE22Sm grown alone (1.8 × 10^8^ CFU/ml), but about two orders of magnitude higher than when RE22Sm was co-cultured with either S4Sm (3.1 × 10^6^ CFU/ml) or *exoP* (2.6 × 10^6^ CFU/ml) at 123 h (Fig. 2c, P < 0.05). These data showed that the *clpX* mutant was not able to inhibit RE22Sm growth or biofilm formation under the tested conditions. While the *exoP* mutant was able to inhibit RE22Sm biofilm formation to the same extent as the wild type S4Sm, the *exoP* mutant showed decreased ability to inhibit RE22Sm planktonic growth.

### Effects of exogenous TDA on the antagonistic activity of the *clpX* mutant

**Fig. 3:**
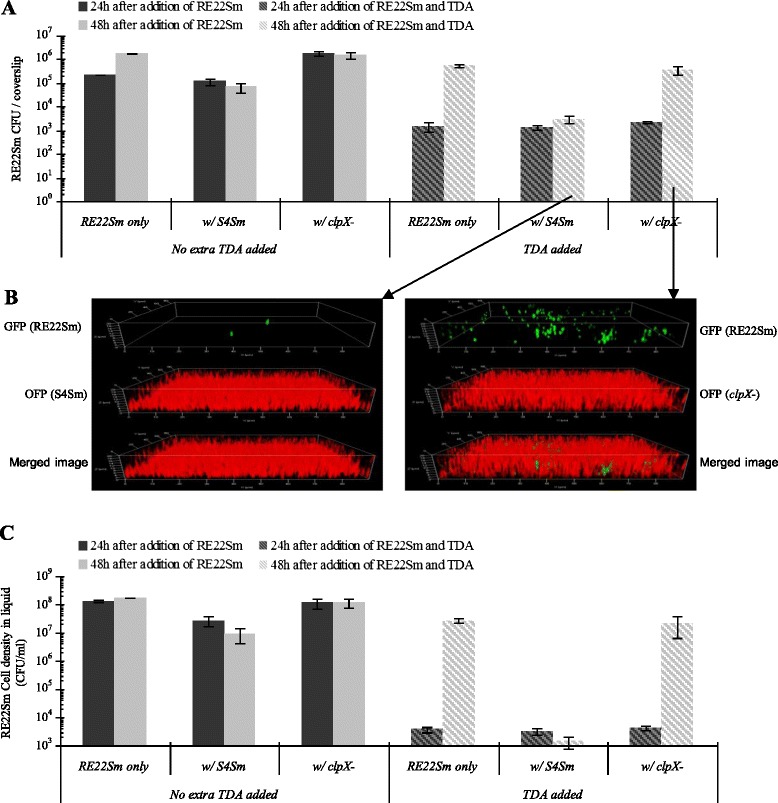
Effects of TDA supplementation on competition assays between *P. inhibens* strains and *V. tubiashii* RE22Sm. **a** Growth of sessile RE22Sm on a glass coverslip in co-culture with either the S4Sm wild type or the *clpX* mutant strain (WZ10) supplemented with or without TDA (10 μg/ml). **b** Single channel and merged confocal microscopy images of mixed biofilm development by OFP-producing strains of *P. inhibens* S4Sm (WZ02) or the *clpX* mutant (WZ12) with the GFP-producing strain of *V. tubiashii* RE22Sm (WZ103) on the surface of a glass coverslip at 48 h after addition of RE22Sm and TDA. **c** Growth of planktonic RE22Sm in co-culture system with either the S4Sm wild type or the *clpX* mutant strain supplemented with or without TDA (10 μg/ml). The data presented are averages of two independent experiments with each experiment done in triplicate. Error bars represent one standard deviation

In order to confirm the relationship between the loss of TDA production and the inability of the *clpX* mutant biofilm to block colonization by the tested pathogens, exogenous TDA (10 μg/ml) or the same volume of distilled water (no extra TDA added) was added to the co-culture system of the *clpX* mutant (TDA deficient) and RE22Sm at the same time as the pathogens. Addition of TDA suppressed biofilm formation (Fig. 3a, by ~10^2^ to 10^3^ fold) and planktonic growth (Fig. 3c, by >10^4^ fold) by RE22Sm cells for 24 h. This was seen in mixed cultures of RE22Sm plus either S4Sm or the *clpX* mutant or in the monoculture of RE22Sm. Further, the number of RE22Sm cells in all three TDA-supplemented cultures, whether as biofilms or planktonic cells, was not statistically different. However, the effects of a single dose of exogenous TDA were transitory. At 48 h, the amount of RE22Sm cells co-cultured with the *clpX* mutant and exogenous TDA increased over 160-fold, and were not significantly different from the values for RE22Sm cultured alone (Fig. 3a and c). The confocal micrographic images of biofilms from 48 h cultures confirmed that more RE22Sm cells (green) were observed in the *clpX* mutant biofilm (with exogenous TDA) than in S4Sm biofilm (Fig. 3b).

### Effects of *V. tubiashii* on growth of *P. inhibens* strains in competition assays

**Fig. 4:**
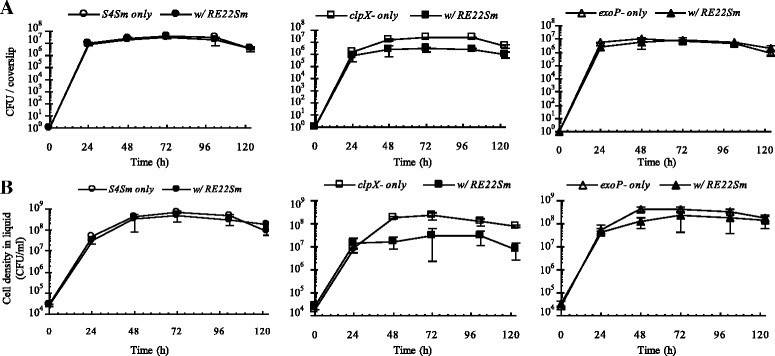
Effects of *V. tubiashii* on the growth of *P. inhibens* strains in a competition assay. *P. inhibens* S4Sm strains were allowed to grow and form biofilms for 24 h before the addition of *V. tubiashii* RE22Sm. The mixed cultures are: S4Sm-OFP with RE22Sm-GFP, *clpX*-OFP with RE22Sm-GFP and *exoP*-OFP with RE22Sm-GFP. **a** Growth of sessile S4 cells (with RE22Sm) in a co-culture system and a monoculture control. **b** Growth of planktonic S4 cells (with RE22Sm) in a co-culture system and a monoculture control. The data presented are averages of two independent experiments with each experiment done in triplicate. Error bars represent one standard deviation

In order to see if *V. tubiashii* would affect the growth of our various *P. inhibens* strains, we compared the growth of *P. inhibens* strains in the presence of *V. tubiashii* with growth in monoculture controls. Growth of wild type S4Sm and the *exoP* mutant were not affected by *V. tubiashii* on the coverslip (Fig. 4a, left and right panels, respectively) or in suspension (Fig. 4b, left and right panels, respectively). In contrast, the growth of the *clpX* mutant was affected by *V. tubiashii*. At each time point tested, the density of the *clpX* mutant (grown with RE22Sm) was lower than that of the monoculture control (Fig. 4a & b, center panels). For example, at 72 h the biofilm density of *clpX* mutant cells grown in the presence of RE22 was 13.2 % of *clpX* mutant cells grown axenically (3.3 × 10^6^ ± 5.3 × 10^5^ CFU/coverslip vs. 2.5 × 10^7^ ± 1.2 × 10^6^ CFU/coverslip, P < 0.05). Similarly, the planktonic cell density of *clpX* mutant cells grown in the presence of RE22Sm was 13.5 % of *clpX* mutant cells grown axenically (3.1 × 10^7^ ± 6.0 × 10^6^ CFU/ml vs. 2.3 × 10^8^ ± 6.9 × 10^7^ CFU/ml, P < 0.05). Additionally, when *V. anguillarum* NB10Sm was co-cultured with either S4Sm or the *exoP* mutant, it did not affect their growth; however, NB10Sm did inhibit the growth of the *clpX* mutant (Additional file 6).

### Effects of co-incubation with *Phaeobacter* strains on pathogen growth and biofilm formation

**Fig. 5:**
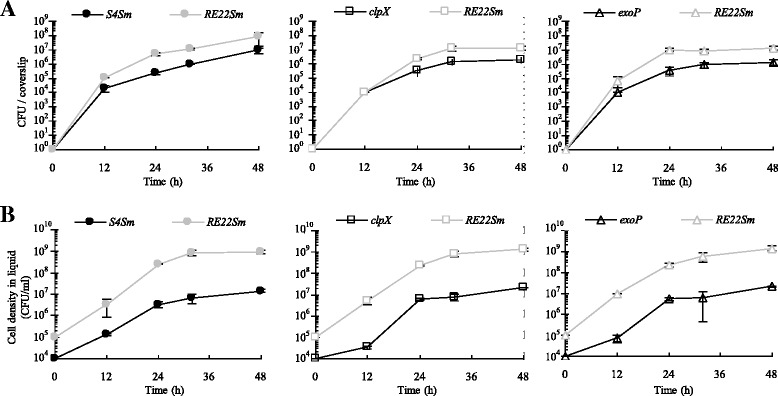
Competition between *P. inhibens* S4Sm strains and *V. tubiashii* RE22 without pre-colonization by *P. inhibens*. The mixed cultures are: S4Sm-OFP with RE22Sm-GFP, *clpX*-OFP with RE22Sm-GFP, and *exoP*-OFP with RE22Sm-GFP. **a** Growth of sessile S4 strains and RE22Sm in a co-culture system. **b** Growth of planktonic S4 strains and RE22Sm in a co-culture system. The data presented are averages of two independent experiments with each experiment done in triplicate. Error bars represent one standard deviation

To determine if *P. inhibens* S4Sm can compete against *Vibrio* pathogens when added at the same time, competition assays were performed (Fig. 5). The amount of RE22Sm cells in the biofilm was ~8.3 × 10^7^ CFU/coverslip at 48 h (Fig. 5a). This was 830-fold more RE22 cells than detected in the biofilm which was pre-colonized with S4Sm for 24 h (1 × 10^4^ CFU/coverslip; Fig. 2b). Similarly, without pre-colonization by the *P. inhibens* mutants (*clpX* or *exoP* mutants) RE22Sm exhibited 10- to 100-fold more cells in the mixed biofilm compared to biofilms formed with pre-colonization by the *P. inhibens* mutants (for *exoP*: the amount of RE22Sm with pre-colonization is ~1 × 10^5^ CFU/coverslip, without pre-colonization ~1.1 × 10^7^ CFU/coverslip; for *clpX*: with pre-colonization ~ 1.2 × 10^6^ CFU/coverslip, without pre-colonization ~ 1.0 × 10^7^ CFU/coverslip) (Fig. 2b, Fig. 5a). Further, when S4Sm was added at the same time as the pathogen, cell density of planktonic RE22Sm (at 48 h) was ~ 9.3 × 10^8^ CFU/ml (Fig. 5b), more than 30-fold higher than the density of RE22Sm observed in the pre-colonized culture (2.8 × 10^7^ CFU/ml) (Fig. 2d). In contrast, pre-colonization with S4Sm was not necessary to antagonize *V. anguillarum* (NB10Sm). In experiments where S4Sm and NB10Sm were inoculated together, NB10Sm was eliminated from both the coverslip biofilm and the liquid culture by 40 to 48 h (Additional file 7). Further, the *exoP* mutant inhibited NB10Sm biofilm formation and growth in suspension almost as well as S4Sm. In contrast, the *clpX* mutant (TDA deficient) exhibited almost no inhibition of either biofilm formation or planktonic growth of NB10Sm, compared to NB10Sm grown alone. These observations are also illustrated by the confocal images of biofilms formed by OFP-tagged *P. inhibens* strains and GFP-tagged NB10Sm (WZ203) cells (Additional file 7).

### Effect of mutations in *clpX* and *exoP* on probiotic activity of *P. inhibens* against *V. tubiashii* in oyster larvae

**Fig. 6:**
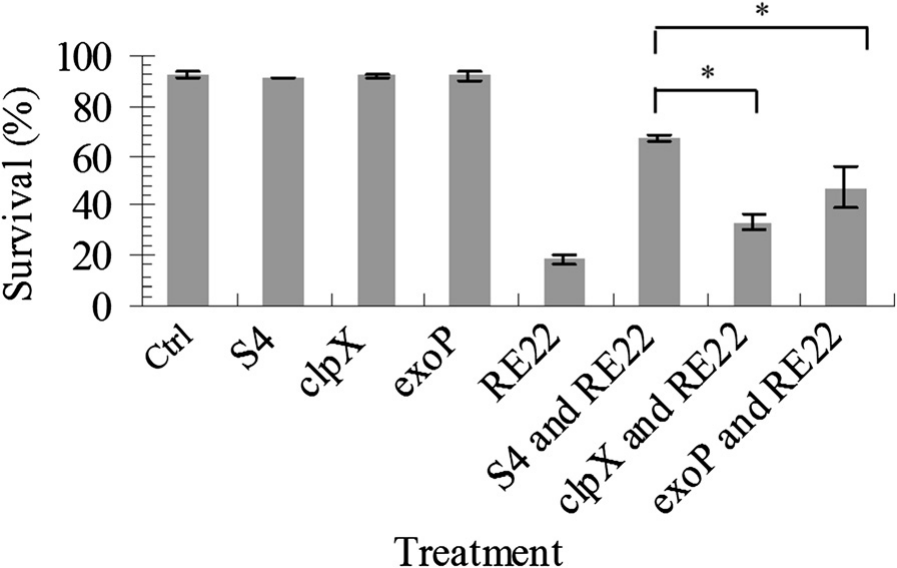
Oyster larvae survival in the presence of *P. inhibens* strains after challenge with *V. tubiashii*. The *P. inhibens* S4Sm strains (1 × 10^4^ CFU/ml) were introduced 24 h before larvae were challenged with *V. tubiashii* RE22Sm (1 × 10^5^ CFU/ml). Oyster larvae treated only with artificial seawater served as control (mock). Oyster larvae survival (% ±SD) was determined 24 h after challenge with RE22Sm Bars marked with an asterisk (*) show significant differences (p <0.05). Representative of at least 3 experiments. Error bars represent one standard deviation

In order to determine if mutations in TDA production or biofilm formation would affect the probiotic activity of S4Sm against *V. tubiashii in vivo*, larval oyster challenge assays were performed as described by Karim et al. [16]. *P. inhibens* mutants showed a significant reduction in their ability to protect larval oysters against *V. tubiashii* challenge compared to wild type S4Sm (Fig. 6). The *clpX* mutant exhibited a >50 % decline in oyster larvae survival compared to S4Sm (S4Sm: 72.4 % ± 1.4 % vs. *clpX*: 35.7 % ± 3.3 %, P < 0.05), while the *exoP* mutant provided almost 70 % of the protection as S4Sm (S4Sm: 72.4 % ± 1.4 % vs. e*xoP*: 50.6 % ± 8.3 %, P <0.05) (Fig. 6). Thus, both *P. inhibens* mutants still provided partial protection. Survival in larvae pretreated with either the *clpX* or *exoP* mutant (35.7 % ± 3.3 % and 50.6 % ± 8.3 %, respectively) was significantly higher than the survival of larvae treated only with RE22 (20.3 % ± 1.9 %, P < 0.05) (Fig. 6).

## Discussion

Several *Phaeobacter* species are known to have probiotic activity and are able to protect fish species against bacterial pathogens [11]. The production of the broad-spectrum antibiotic, tropodithietic acid (TDA), is regarded as one of the major factors contributing to probiotic activity against *V.anguillarum* infection in turbot and cod [11]. We recently reported that the new isolate *P. inhibens* S4Sm protects the Eastern oyster (*Crassostrea virginica*) from infection by two oyster pathogens, *V. tubiashii* and *R. crassostreae* [16]. In this report, we dissect the roles of TDA biosynthesis and biofilm formation in promoting probiotic activity by *P. inhibens* S4Sm, showing that both mechanisms are involved.

Although the TDA biosynthetic pathway has not been fully elucidated, many of the genes required for the formation of TDA and much of the pathway have been discovered [21, 23, 24]. One gene reported to be involved in TDA biosynthesis is *clpX* (encoding ClpX) [21]. ClpX is an AAA+ ATPase that functions as an unfoldase chaperon for ClpP (ATP-dependent protease) and with ClpP forms the multimeric ClpXP protease [25]. An insertional mutation in the *clpX* gene specifically blocked the biosynthesis of TDA in S4Sm (Fig. 1a) without affecting biofilm formation (Table 1) or growth (Additional file 4). Further, the effects of mutations to *clpX* upon cell physiology are subtle and diverse [26–28]. In contrast, mutations in *tdaA*, *tdaB*, and *tdbD*, all block TDA biosynthesis and also affect biofilm formation in S4Sm. The mechanism by which ClpX affects TDA production is still unknown. Additionally, the reasons why mutations in *tdaA*, *tdaB*, and *tdbD* decrease biofilm formation, as well as TDA biosynthesis, are not understood, and are not the focus of this study.

The *clpX* TDA deficient mutant was unable to inhibit *V. tubiashii* growth in either liquid or as a biofilm on a glass coverslip (Fig. 2); however, when cultures were supplemented with TDA, the growth of planktonic *V. tubiashii* growth was inhibited (Fig. 3). It is well known that organisms in biofilms are more resistant to antibiotics than when suspended in liquid [29]. This is consistent with our data showing that TDA antibiotic activity was more potent against planktonic RE22 cells than towards RE22 cells living in a biofilm. These data, in which the effect of the wild type was restored by adding TDA to the *clpX* antibiotic activity mutant, strongly suggest that the loss of TDA production is responsible for the defect in antagonistic activity in the *clpX* mutant. Further, 48 h after the addition of TDA into the co-culture the inhibitory effect of TDA disappeared, likely due to instability of TDA over time or metabolism by *V. tubiashii*. Except for the loss of TDA synthesis, the *clpX* mutant exhibited no other defects in growth or biofilm formation compared to the S4Sm wild type when grown in pure culture (Additional file 5). The results reported here confirm the role of TDA as an antibiotic promoting probiotic activity of *Phaeobacter* species described previously by D’Alvise et al. [11] in another host-pathogen system. It is interesting to note that the growth of the *clpX* mutant is depressed by RE22 (Fig. 2), suggesting that TDA production allows *P. inhibens* to compete with faster growing species for available nutrients.

*P. inhibens*, a member of the abundant marine Roseobacter clade, is known to be an excellent colonizer of environmental surfaces [23]. While no study of the effects of biofilm formation on the probiotic mechanism of *Phaeobacter* has been reported, it is interesting to note that Prol Garcia et al. (2014) recently reported that biofilm formation is not a prerequisite for TDA formation in *P. inhibens*. In that study, the authors, using Tn5 transposon mutagenesis, identified 22 TDA-positive mutants with defects in biofilm formation. Among classes of genes identified as contributing to biofilm formation were those involved in exopolysaccharide formation. In our study, the *exoP* gene was identified in S4Sm (using RAST [30]) as an exopolysaccharide biosynthesis gene, which is thought to be involved in biofilm formation. Mutation of *exoP* resulted in a large decrease in biofilm formation (Table 1), and exhibited no other defects in growth or TDA formation (Fig. 1c and Table 2). Thus, our observations correspond to those reported by Prol Garcia et al. (2014) that biofilm formation is not a prerequisite for TDA production and also that mutation of a gene involved with exopolysaccharide production can affect biofilm formation. While the *exoP* mutant was modestly defective in its ability to inhibit *Vibrio* species in competition assays (Figs. 2 & 5) it did exhibit significantly decreased probiotic activity in the oyster challenge assay against *V. tubiashii* (Fig. 6), these declines were less than those seen in the *clpX* mutant. We suggest that while in the *in vitro* (glass coverslip) model the *exoP* mutant forms much less biofilm than the wild type, enough TDA accumulates to inhibit RE22 to levels near those caused by wild type cells. However, in the *in vivo* oyster challenge model, the reduced biofilm of the *exoP* mutant results in decreased TDA production that is diluted by the larger volumes of the system and the feeding activity of the oyster larvae causing less inhibition of RE22. These data suggest that biofilm formation contributes to the probiotic activity of S4Sm. Biofilms may contribute to probiotic activity in two ways. First, biofilms would allow *P. inhibens* to physically occupy potential sites of colonization and prevent the oyster pathogens from gaining access to the oyster. Second, the formation of an extensive biofilm with cells at high density may induce the production of TDA [31]. A more extensive biofilm would produce more TDA and, therefore, more effectively inhibit the ability of pathogens to infect the oyster host.

As a broad spectrum antibiotic TDA inhibits the growth of several marine pathogens [32]. However, in the ocean environment TDA will be rapidly diluted once it is secreted. We suggest that *P. inhibens* requires both TDA production and biofilm formation for effective probiotic activity. The biofilm matrix creates a microenvironment within which TDA can accumulate to reach concentrations high enough to inhibit pathogens. In the absence of TDA, a *P. inhibens* biofilm does not eliminate pathogens and provides only modest protection against disease. Further, *P. inhibens* growing with a diminished biofilm also exhibits significantly reduced probiotic activity probably due to the decreased mass of cells producing TDA and the increase in available sites for pathogens to colonize. Our data indicate that maximum probiotic activity requires both TDA production and biofilm formation.

Karim et al. [16] reported that oyster larvae were best protected when *P. inhibens* S4Sm was added 24 h prior to challenge by either of the two oyster pathogens, *V. tubiashii* and *R. crassostreae*. The data presented in this report is consistent with those previous observations and reveal that pre-colonization of a surface by S4Sm is more effective than co-incubation at inhibiting *V. tubiashii* RE22 from either colonizing the glass coverslip surface or from growing planktonically (Figs. 2 and 5). One potential reason for this need for a 24 h pre-treatment is the rapid generation time of *Vibrio* species in YP30 (at 27 °C, with shaking), which is less than 1 h (*V. tubiashii* is ~0.53 h, *V. anguillarum* is ~0.89 h), while the doubling time for *P. inhibens* S4Sm is ~3.1 h. Successful probiotic activity by S4Sm may be dependent upon growth rate and having enough TDA producing cells in the biofilm to successfully antagonize and out-compete the oyster pathogens. Interestingly, we show in our study that *V. anguillarum* cells are more sensitive to TDA than are *V. tubiashii* cells, and that, while pre-colonization of surfaces by S4Sm was required to prevent the colonization of coverslips by *V. tubiashii,* it was not required to prevent the colonization by *V. anguillarum*. Consistent with these observations, D’Alvise et al. [11] showed that it was not necessary for *P. gallaeciensis* to precolonize the wells containing cod larvae in order to antagonize *V. anguillarum* and significantly reduce cod larvae mortalities. Our experiments indicate differences between *Vibrio* species on how they interact with the S4Sm probiotic. Interestingly, precolonization with RE22 reduces the ability of S4 and mutants to grow & colonize glass cover slips and to grow planktonically (Fig. 5), suggesting that RE22 is able to modulate the probiotic activity of S4Sm through negative impacts on the ability to grow and/or colonize surfaces.

## Conclusions

The results presented in this study demonstrate that both TDA production and biofilm formation contribute to the probiotic activity of *P. inhibens* S4Sm. Specifically blocking TDA production by mutation of the *clpX* gene resulted in a significant decline in probiotic activity as determined by coverslip colonization assay or by survival of oyster larvae challenged by *V. tubiashii* RE22. While reducing biofilm formation by mutation of the *exoP* gene also resulted in a significant decline in probiotic activity as determined by survival of oyster larvae challenged by *V. tubiashii* RE22, but only a modest decline as measured by coverslip colonization assay. It is possible that biofilm formation contributes to probiotic activity in two ways: 1) occupying potential colonization sites and 2) increasing cell density-dependent induction of TDA biosynthesis. Future investigation will examine these possibilities.

## Methods

### TDA purification, identification and detection

TDA was produced and extracted using a modified method of Bruhn *et al.* [13]. *P. inhibens* S4Sm was cultured in 7 x 1 L volumes of YP30 culture medium at 27 °C with shaking at 175 rpm. After 96 h, the cells were pelleted by centrifugation at 10,000 rpm for 10 min. The resulting culture supernatants were acidified to pH 3 with formic acid (FA) and extracted with acidified (0.1 % FA) ethyl acetate. The organic fraction was concentrated *in vacuo* to yield 0.673 g of crude extract. The extract was fractionated using C18 flash chromatography (Redisep Rf high performance gold 30 g hp combiflash column; linear gradient elution 5 % - 100 % CH_3_OH in H_2_O, 0.1 % FA, 35 ml/min, 45 min). Fractions containing TDA (t_R_ = 15 min) were further purified by reversed-phased HPLC (Xterra 5 μm C18 100 x 3.0 mm column, 0.5 ml/min, 5 % to 100 % CH_3_OH in H_2_O over 24 min). Pure TDA (10 mg) was identified based on comparison of ^1^H NMR (Varian 500 MHz spectrometer) and mass spectral data in comparison to previously reported values (Additional files 1, 2 and 3) [22]. All assays were conducted with purified TDA from *P. inhibens* S4Sm.

Culture supernatants from *P. inhibens* wild type and mutant strains were analyzed by HPLC for the presence of TDA. *P. inhibens* strains were cultivated in 50 ml YP30 broth until stationary phase (2 × 10^9^ CFU/ml). Cells were pelleted by centrifugation (5000 × *g*, 10 min) and extracted as described above. The resulting organic extracts were reconstituted as 10 mg/ml solutions in methanol. Chromatography was performed on a Hitachi LaChromUltra UHPLC equipped with a Fortis C18 UHPLC Column (1.7 μm, 2.1 x 50 mm). Method: 0.25 ml/min flow rate, 5 % CH_3_OH in H_2_O (both acidified with 0.1 % FA) for 1 min, linear gradient to 100 % CH_3_OH over 6.2 min, 100 % CH_3_OH for 2 min.

### Minimum inhibitory concentrations of TDA against *V. anguillarum*, *V. tubiashii,* and *R. crassostreae*

The minimal inhibitory concentrations (MIC) of TDA against selected marine pathogens were determined using a broth dilution method in microtiter plates [33]. Overnight bacterial cultures were diluted to 10^5^ CFU/ml in YP30 and treated with serial dilutions of pure TDA. After 24 h incubation at 27 °C, MICs were determined as the lowest concentration where there was no visible growth. Two independent experiments were done and each independent experiment had three replicates.

### Bacterial strains, plasmids, and growth conditions

All bacterial strains and plasmids used in this report are listed in Table 3. *P. inhibens* strains were routinely grown in yeast extract (0.5 %)-peptone (0.1 %) broth plus 3 % sea salts, pH 7.6 (YP30) [16], supplemented with the appropriate antibiotic, in a shaking water bath (175 rpm) at 27 °C. Overnight cultures (2 × 10^9^ CFU/ml) of *P. inhibens*, grown in YP30, were harvested by centrifugation (8000 × *g*, 2 min) and the pelleted cells were washed twice with nine-salt solution (NSS) [34]. Washed cells were resuspended to appropriate cell densities in experimental media. Cell densities were estimated by optical density at 600 nm (OD_600_) and more accurately determined by serial dilution and spot plating. Specific conditions for each experiment are described in the text. *Escherichia coli* strains were routinely grown in Luria-Bertani broth plus 1 % NaCl (LB10) [35]. *Vibrio anguillarum* strains were routinely grown in LB20 at 27 °C [36]. *V. tubiashii* and *R. crassostreae* strains were routinely grown in YP30 at 27 °C [16]. Antibiotics were used at the following concentrations: streptomycin, 200 μg/ml (Sm^200^); ampicillin, 100 μg/ml (Ap^100^) for *E. coli* and *Vibrio* strains; chloramphenicol, 20 μg/ml (Cm^20^) for *E. coli* and 5 μg/ml (Cm^5^) for *P. inhibens* and *Vibrio* strains; kanamycin, 50 μg/ml (Km^50^) for *E. coli* strains and 200 μg/ml (Km^200^) for *P. inhibens*; and tetracycline, 15 μg/ml (Tc^15^) for *E. coli* and 1 μg/ml (Tc^1^) for *V. anguillarum*. Frozen stocks in glycerol were maintained at −74 °C and cultures were routinely identified by phenotypic and genotypic characteristics.

**Table 3:** Bacterial strains and plasmids used in this study

Strains or plasmids	Description	Resistance	Reference
*P. inhibens*			
S4	Previously *Phaeobacter sp.* S4; wild type isolate from the inner shell of oysters		Karim et al., 2013
S4Sm	Spontaneous Sm^r^ mutant of S4	Sm^r^	this study
WZ10	*clpX* insertional mutant of S4Sm	Sm^r^ Cm^r^	this study
WZ11	*clpX+*, clpX *in trans* complement of WZ10	Sm^r^ Cm^r^ Ap^r^	this study
WZ20	*exoP* insertional mutant of S4Sm	Sm^r^ Cm^r^	this study
WZ21	*exoP+*, exoP *in trans* complement of WZ20	Sm^r^ Cm^r^ Ap^r^	this study
WZ02	S4Sm (pRhokHi-2-*ofp*)	Sm^r^ Cm^r^ Km^r^	this study
WZ12	*clpX*, WZ10 (pRhokHi-2-*ofp*)	Sm^r^ Cm^r^ Km^r^	this study
WZ22	*exoP*, WZ20 (pRhokHi-2-*ofp*)	Sm^r^ Cm^r^ Km^r^	this study
*V. tubiashii*			
RE22	Wild type isolate from oyster larvae		Estes et al., 2004
RE22Sm	Spontaneous Sm^r^ mutant of RE22	Sm^r^	this study
WZ103	RE22Sm (pRhokHi-2-*gfp*)	Sm^r^ Ap^r^	this study
*V. anguillarum*			
NB10	Wild type, serotype O1, clinical isolate from the Gulf of Bothnia		Norqvist et al., 1989
NB10Sm	Spontaneous Sm^r^ mutant of NB10	Sm^r^	this study
WZ203	NB10Sm (pSUP202P-P*flaB*-*gfp*)	Sm^r^ Ap^r^ Tet^r^	this study
*R. crassostreae*			
CV919-312^T^	Wild type isolate from a JOD-affected oyster		Boettcher et al., 1999
CV919Sm	Spontaneous Sm^r^ mutant of CV919-312 T	Sm^r^	this study
*E. coli*			
Sm10	*thi thr leu tonA lacY supE recA* RP4-2 Tc::Mu::Km (λpir)	Km^r^	Simon et al., 1983
S100	Sm10 harboring pNQ705-1		this study
WQ10	Sm10 harboring pNQ705-*clpX*		this study
WQ20	Sm10 harboring pNQ705-*exoP*		this study
WB01	Sm10 harboring pBBR1MCS4		this study
WB11	Sm10 harboring pBBR1MCS4-*clpX*		this study
WB21	Sm10 harboring pBBR1MCS4-*exoP*		this study
S122	Sm10 harboring pSUP202P-*gfp*(ORF)		this study
S136	Sm10 harboring pSUP202P-P*flaB*-*gfp*		this study
W900	Sm10 harboring pRhokHi-2-FbFP		this study
WR03	Sm10 harboring pRhokHi-2-*gfp*		this study
WR02	Sm10 harboring pRhokHi-2-*ofp*		this study
W901	Sm10 harboring pmOrange		this study
Plasmids			
pNQ705-1	Cm^r^; suicide vector with R6K origin		Mcgee, 1996
pNQ705-*clpX*	Cm^r^; derivative from pNQ705-1 for *clpX* insertional mutant		this study
pNQ705-*exoP*	Cm^r^; derivative from pNQ705-1 for *exoP* insertional mutant		this study
pBBR1MCS4	Ap^r^; derivative from pBBR1MCS (a broad-host-range cloning vector)		Kovach et al., 1995
pBBR1MCS4-*clpX*	Apr; derivative from pBBR1MCS4 for *clpX in trans* complement		this study
pBBR1MCS4-*exoP*	Apr; derivative from pBBR1MCS4 for *exoP in trans* complement		this study
pBS(gfp)-Pcampy	Template for *gfp* ORF PCR amplification		Eggers et al., 2004
pCE320(gfp)-PflaB	Template for *PflaB* PCR amplification		Eggers et al., 2004
pSUP202P	Ap^r^ Cm^r^ Tc^r^; broad host shuttle vector		Simon et al., 1983
pSUP202P-*gfp*(ORF)	Ap^r^ Tc^r^; derivative from pSUP202 for GFP tagging		this study
pSUP202P-P*flaB*-*gfp*	Ap^r^ Tc^r^; derivative from pSUP202 for GFP tagging		this study
pRhokHi-2-FbFP	Cm^r^ Km^r^; derivative from pBBR1MCS (a broad-host-range cloning vector) with promoter PaphII		Piekarski et al., 2009
pRhokHi-2-*gfp*	Cm^r^ Km^r^; derivative from pRhokHi-2-FbFP with *gfp* under the control of P*aphII*		this study
pmOrange	Template for *ofp* ORF PCR amplification		Clontech Laboratories, Inc.
pRhokHi-2-*ofp*	Cm^r^Km^r^; derivative from pRhokHi-2-FbFP with *ofp* under the control of P*aphII*		this study

### Insertional mutagenesis

Insertional mutagenesis by homologous recombination was used to create interruptions within specific genes using a modification of the procedure described by Milton and Wolf-Watz [37, 38]. Primers (Table 4) were designed to amplify specific *Phaeobacter* genes based on homologous sequences from *P. inhibens* 2.10 (GenBank accession No.CP002972.1) *(Phaeobacter* 2.10 was reclassified into *P. inhibens* from *P. gallaeciensis* in 2013 [39]). A fragment of the selected gene was PCR amplified, then digested with SacI and XbaI restriction enzymes, and the DNA fragments separated on a 1 % agarose gel. The gel-purified PCR fragment was ligated into the suicide vector pNQ705 after digestion with SacI and XbaI and the ligation mixture was introduced into *E. coli* Sm10 (λ pir) by electroporation (0.2 cm cuvette, 2.5 kV, 200 Ω, 25 μF) with Bio-Rad Gene Pulser II. Recombinant plasmids were confirmed by both PCR amplification and sequencing. The mobilizable suicide vector was transferred from *E. coli* Sm10 (λ pir) into S4Sm by conjugation. Transconjugants were selected by utilizing the chloramphenicol resistance gene located on the suicide plasmid. The incorporation of the suicide vector into the gene of interest was confirmed by PCR analysis and DNA sequencing of the mutated genes [37].

**Table 4:** Primers used in this study

Primer	Sequence (5′ to 3′, underlined sequences are engineered restriction sites)	Description
pw30	GTATTAGAGCTCATCGCACTGCTTCTTGAGGT	For *tdaA* insertional mutation, forward, with *Sac*I site
pw31	CGACTATCTAGAGATGATTGGGTCCTTTGCAC	For *tdaA* insertional mutation, reverse, with *Xba*I site
pw32	GTATTAGAGCTCAGCAGCCATGAATAGCCTGT	For *tdaB* insertional mutation, forward, with *Sac*I site
pw33	CGACTATCTAGAGGGTATCGGATTTCGGATTT	For *tdaB* insertional mutation, reverse, with *Xba*I site
pw36	GTATTAGAGCTCATCTTTGGCTCCATCGACAT	For *tdbD* insertional mutation, forward, with *Sac*I site
pw37	CGACTATCTAGAGCACATTGTTGGGAAACTGA	For *tdbD* insertional mutation, reverse, with *Xba*I site
pw108	GAAGAGCTCGGACGACTATGTGATTGGTCAGGC	For *clpX* insertional mutation, forward, with *Sac*I site
pw109	GGGTCTAGACGACGTTATATTCCGACGCCTGCA	For *clpX* insertional mutation, reverse, with *Xba*I site
pw153	GTATTAGAGCTCGAGCATAACCGCTTTGCCCGCCGCCCA	For *exoP* insertional mutation, forward, with *Sac*I site
pw154	CGACTATCTAGACCATGCTGAGTGCAAGGTTGACGGCGG	For *exoP* insertional mutation, reverse, with *Xba*I site
pw127	GCATTAGAGCTCGTCAGATTGGCCGAAGCCCCTTTT	For *clpX in trans* complement, forward, with *Sac*I site
pw128	CGGCTATCTAGACGAACTCACCACCTGAGGAGATACGT	For *clpX in trans* complement, reverse, with *Xba*I site
pw166	GTATTAGAGCTCCCCGTCCGATGTGTCAAAATAGGT	For e*xoP in trans* complement, forward, with *Sac*I site
pw165	CGTCTTTCTAGAGGTGCCTGCGGTCATCACCATGAC	For *exoP in trans* complement, reverse, with *Xba*I site
pwGFP-F	GCGGTACATATGTAAGGAGGAAAAACATATG	For amplification of *gfp* ORF, forward, with *Nde*I site
pwGFP-R	CTATATGGATCCCAGATCTATTTGTATAGTTCATCCA	For amplification of *gfp* ORF, reverse, with *BamH*I site
Pm113	GGTACCTGTCTGTCGCCTCTTGT	For amplification of PflaB, forward, with *Kpn*I site
Pm114	GGTACCATATCATTCCTCCATGAT	For amplification of PflaB, forward, with *Kpn*I site
pwmO-F	GCGGTACATATGATGGTGAGCAAGGGCGAGGAGAAT	For amplification of *ofp* ORF, forward, with *Nde*I site
pwmO-R	CTATATGGATCCCTTGTACAGCTCGTCCATGCCGCC	For amplification of *ofp* ORF, reverse, with *Bam*HI site

### Complementation of mutants

*P. inhibens* mutants were complemented by cloning the target gene fragment into the shuttle vector pBBR1MCS4 (GenBank accession No. U25060), using a modification of the method by Rock and Nelson [40]. Primers (Table 4) were designed with a SacI or XbaI site added to the 5′ end of the appropriate primer. The primer pair was used to amplify the entire gene plus ∼ 500 bp of the 5′ and 3′ flanking regions from genomic DNA sequences of *P. inhibens* 2.10 (GenBank accession No.CP002972.1). The resulting amplicon was ligated into the pBBR1MCS4 plasmid after digestion with SacI and XbaI and the ligation mixture introduced into *E. coli* Sm10 (λ pir) by electroporation with Bio-Rad Gene Pulser II. Transformants were selected on LB10-Amp100 agar plates and the recombinant plasmids confirmed by both PCR amplification and sequencing. The complementing plasmid, pBBR1MCS4-*clpX* or pBBR1MCS4-*exo*P, was transferred from *E. coli* Sm10 into *clpX* or *exoP* mutants by conjugation using the procedures described previously [37, 41]. The transconjugants were confirmed by PCR amplification.

### Fluorescence tagging of *P. inhibens* strains and *Vibrio* species

*P. inhibens* strains were tagged by pRhokHi-2-OFP and *V. tubiashii* was tagged by pRhokHi-2-GFP. The orange fluorescence protein gene (*ofp*) and the green fluorescence protein gene (*gfp*) were PCR amplified by using the appropriate primer pair (Table 4) designed according to the sequence of pmOrange vector (Clontech) and pSUP202p/P*flaB*-*gfp* vector. The PCR product was digested with NdeI and BamHI restriction enzymes and the DNA fragments separated on a 1 % agarose gel. Subsequently, the gel-purified *ofp* or *gfp* PCR fragment was ligated into pRhokHi-2 after digestion with NdeI and BamHI and the ligation mixture was introduced into *E. coli* Sm10 (λpir) by electroporation with Bio-Rad Gene Pulser II. Transformants were selected on LB10-Cm^20^ agar plates. All plasmids were transferred from *E. coli* SM10 to recipient strains of *P. inhibens* S4Sm, *V. tubiashii* RE22Sm, *R. crassostreae* CV919Sm, and *V. anguillarum* NB10Sm using the method described previously by Mou et al. [41]. The transconjugants were confirmed by fluorescence microscopy.

### Biofilm formation

Biofilm formation was assessed using a modification of the crystal violet (CV) staining method [19]. Bacteria were grown for 2 days in YP30 (27 °C with shaking) to stationary phase (2 × 10^9^ CFU/ml). Cells (2 μl) were transferred into 2 ml of fresh YP30 broth in 30 mm × 100 mm borosilicate (Pyrex) glass culture tubes containing 2 ml YP30 broth and allowed to grow at 27 °C without shaking. When sampling, the liquid culture was discarded and each tube rinsed twice with NSS to remove loosely attached cells. The biofilm attached to the test tube wall was stained with 2 ml of CV solution (0.2 %) for 20 min at room temperature. Unbound dye was removed with two washes of NSS. The bound dye was eluted with 95 % (vol/vol) ethanol for 30 min and then the amount of eluted crystal violet was measured by spectroscopy at 580 nm using a VERSA-MAX microplate reader.

### Inhibition zone assay

Anti-bacterial activity of *P. inhibens* strains was measured by a growth inhibition assay using *V. anguillarum*, *V. tubiashii*, and *R. crassostreae* as the target organisms. An aliquot (100 μl) from a stationary phase overnight culture of the appropriate *Vibrio* or *R. crassostreae* culture (2 × 10^9^ CFU/ml) was spread onto YP30 agar plates, then 10 μl of a 2-day-old culture (2 × 10^9^ CFU/ml) of a *P. inhibens* strain was spotted in triplicate onto the pathogen cell lawn. After incubation at 27 °C for 24 h, the level of antibacterial activity was determined by the diameter of the inhibition zone around the *P. inhibens* colonies.

### *P. inhibens* culture supernatant killing assay

In order to determine the bactericidal activity of culture supernatants, *P. inhibens* strains were grown for 2 days in YP30 (27 °C with shaking). Cultures were centrifuged (8000 × *g*, 10 min) and filtered through 0.2 μm pore membrane filters to collect filter sterilized cell-free supernatants. Overnight cultures of *V. anguillarum* (NB10Sm) cells (2 × 10^9^ CFU/ml) were then serially diluted in filter sterilized, cell-free *P. inhibens* culture supernatant obtained from the various strains of *P. inhibens* or NSS, and then spotted (10 μl/spot of diluted *V. anguillarum* cells) in triplicate onto YP30 plates. All experiments were repeated twice. Killing percentage was calculated as follows: Killing % = [(no. of colonies in NSS control) – (no. of colonies in S4 supernatant treated)/(no. of colonies in NSS control)] × 100.

### Glass coverslip colonization competition assay between *P. inhibens* strains and *V. tubiashii* WZ103 or *V. anguillarum* WZ203

This assay was performed using a modification of establishment and invasion of pre-established biofilms method [42]. For all competition experiments, *P. inhibens* strains (S4Sm, *clpX* mutant and *exoP* mutant) were grown for 2 days in YP30 (27 °C with shaking) to stationary phase. Cells were harvested by centrifugation, washed twice in NSS, resuspended in fresh YP30, and then transferred into 6-well plates (Costar, Tewksbury MA). Each well contained a glass coverslip, 4 ml YP30 broth supplemented with streptomycin, and was inoculated with the appropriate *P. inhibens* strain (WZ02, WZ12, or WZ22) tagged with orange fluorescence protein (OFP) (final concentration ~1 × 10^4^ CFU/ml). For experiments examining the effects of pretreatment with *P. inhibens*, after 24 h incubation at 27 °C with no shaking (pretreatment with *P. inhibens*) all coverslips were washed twice with NSS. Each coverslip was transferred into a fresh well containing 4 ml of YP30 broth supplemented with streptomycin plus the green fluorescence protein (GFP)-tagged *V. tubiashii* WZ103 or GFP-tagged *V. anguillarum* WZ203 (final concentration ~1 × 10^5^ CFU/ml). After another 24 h incubation at 27 °C with no shaking, all coverslips were removed, washed twice on a rotary shaker (LAB-LINE instrument, Inc.) for 2 min (200 rpm) with NSS, and then transferred into clean wells with fresh YP30 broth and allowed to incubate as before. Two coverslips were removed at each sampling time (24, 48, 72 h). One was used for determination of the cell density of the strains on the coverslip; the second one was used for confocal imaging. Glass coverslips were washed with NSS twice on a rotary shaker for 2 min. After draining excess water, coverslips used for confocal imaging were placed on depression slides and cells on the upside of coverslip were wiped off with Kimwipes™. Coverslips used for CFU determinations were immersed in 50 ml plastic tubes containing 10 ml NSS and glass beads (0.5 g, 1 mm), then vortexed for 1 min. Cell densities (CFU/ml) in the wells or suspended from the coverslip were determined by serial dilution and spot plating. Appropriate antibiotics were used for selection of bacteria (see Table 3 for antibiotic resistances for each strain). For experiments without pretreatment with *P. inhibens*, all procedures were identical to those described above except that GFP-tagged *V. tubiashii* WZ103 or *V. anguillarum* WZ203 were added at the same time as OFP-tagged *P. inhibens*. In the *V. anguillarum* competition experiments, both *P. inhibens* and *V. anguillarum* were inoculated at ~10^6^ CFU/ml.

### Effects of TDA supplementation on pathogen growth in a co-culture system containing the *clpX* mutant and a *Vibrio* species

OFP-tagged *P. inhibens* strains (S4Sm, *clpX* mutant) grown for 2 days in YP30 (27 °C with shaking) to stationary phase, cells were transferred into 6-well plates. Each well was inoculated with the appropriate OFP-tagged *P. inhibens* strain (initial concentration at ~ 10^4^ CFU/ml) in 4 ml of YP30 broth supplemented with the appropriate antibiotic and one glass coverslip. After 24 h incubation (pre-treatment with *P. inhibens*), all coverslips were washed twice in NSS. Each coverslip was transferred into a clean well containing 4 ml YP30 broth and either GFP-tagged *V. anguillarum* WZ203 or *V. tubiashii* WZ103 at a concentration of ~ 10^5^ CFU/ml plus TDA (5 μg/ml for *V. anguillarum* WZ203 or 10 μg/ml for *V. tubiashii* WZ103; based on calculated MIC). The biofilms on the coverslips were imaged as described below and cell densities were determined as described above.

### Laser confocal scanning microscopy

Laser confocal scanning microscopy was performed in the Rhode Island Genomic Sequencing Center using a Zeiss AxioImager 2 microscope equipped for digital image acquisition with a Zeiss AxioCam HRc high-resolution camera and for laser scanning microscopy with a Zeiss LSM 700 confocal module. The confocal module is equipped with four diode lasers with excitation lines at 405, 488, 555, and 639 nm and utilizes the Zeiss ZEN 2011 software.

### Challenge trials

Oyster larvae (n = 21–28 per well, veliger stage, ~0.060–0.150 mm in diameter) were placed in wells of a 6-well plate containing 5 ml of sterile filtered seawater (28 psu). Overnight cultures of *P. inhibens* strains grown in YP30 (~10^9^ CFU/ml) were added to a final concentration of ~10^4^ CFU/ml. Plates were incubated at 20 °C for 24 h with shaking. Water was changed and *V. tubiashii* RE22 was added at a concentration of ~10^5^ CFU/ml in seawater and incubated for an additional 24 h before counting living and dead oysters. Oyster larvae treated only by artificial seawater served as control. The survival rate was calculated by using the formula: *Survival rate (%) = 100 x (number of live larvae/total number of larvae).* These experiments were run at least 2 times in triplicate [16]. As invertebrates, oysters are exempt from approval from the University of Rhode Island Institutional Animal Care and Use Committee.

### Statistical analysis

Data are expressed as means ± standard deviation (SD). Two-tailed, unpaired Student’s *t* tests were used for statistical analyses for all experiments, and *P* values of <0.05 were considered statistically significant.

## Additional files

Additional file 1:
**A) Electrospray ionization MS of purified TDA, structure shown on right, in the positive ion mode.** The spectrum shows expected losses of H2O and CO2H. (PDF 64 kb) Additional file 2:
**1H NMR spectrum (500 MHz, C6D6) for purified TDA.** The spectrum is expanded to show the aromatic region of interest. Spectrum was referenced to residual benzene resonance at 7.16 ppm. (PDF 110 kb) Additional file 3:
**1H NMR spectroscopic data of purified TDA from** ***P. inhibens***. Comparison is provided to data published by Liang (2003). (PDF 7 kb) Additional file 4:
**A) Reversed-phase HPLC chromatograms of ethyl acetate extracts from** ***Phaeobacter*** **S4 mutant strains.** B) Quantification of biofilm formation by measuring OD580 of crystal violet dye attached to the cells forming biofilms on glass tubes at 27 °C under static condition. The data presented are average of two independent experiments and each independent experiment has three replicates. Error bars represent one standard deviation. (PDF 83 kb) Additional file 5:
**Growth curve of** ***Phaeobacter*** **S4 strains under different conditions (static vs. shaking).** Overnight culture of *Phaeobacter* cells were grown in YP30 media and then back-diluted into fresh YP30 1:1000 dilution. Samples were taken at the indicated times and OD600 value were measured by a spectroscopy. Error bars represent one standard deviation. (PDF 340 kb) Additional file 6:
**Effects of** ***V. anguillarum*** **NB10 on the growth of** ***P. inhibens*** **strains in a competition assay without pre-colonization by** ***P. inhibens*** **.** The mixed cultures are: S4Sm-OFP with NB10Sm-GFP, *clpX*-OFP with NB10Sm-GFP and *exoP*-OFP with NB10Sm-GFP. Colonization and initial cell densities were as described in the Materials and Methods. A) Growth of sessile *P. inhibens* cells (with NB10Sm) in a co-culture system and a monoculture control. B) Growth of planktonic *P. inhibens* cells (with NB10Sm) in a co-culture system and a monoculture control. The data presented are average of two independent experiments and each independent experiment has three replicates. Error bars represent one standard deviation. (PDF 362 kb) Additional file 7:
**Competition assay between** ***P. inhibens*** **S4Sm strains and** ***V. anguillarum*** **NB10Sm without pre-colonization by** ***P. inhibens*** **.** The mixed cultures are S4Sm-OFP with NB10Sm-GFP, *clpX*-OFP with NB10Sm-GFP and *exoP*-OFP with NB10Sm-GFP. A) Single channel and merged confocal microscopy images of mixed biofilm development by OFP-producing strains (S4Sm, *clpX* mutant or *exoP* mutant) and GFP-producing *V. anguillarum* NB10Sm-GFP on the surface of glass coverslip at 48 h. The data presented is from a representative experiment of two independent experiments. B) Growth of sessile NB10Sm in co-culture system with different *Phaeobacter* strains. C) Comparison of growth of sessile *V. anguillarum* (NB10Sm) in different co-culture system and monoculture control. D) Growth of planktonic *V. anguillarum* (NB10Sm) in co-culture system with different *Phaeobacter* strains. E) Comparison of growth of planktonic *V. anguillarum* (NB10Sm) in different co-culture system and monoculture control. The data presented are average of two independent experiments and each independent experiment has three replicates. Error bars represent one standard deviation. (PDF 1811 kb)
