# Supplementary material for: Contributions of tropodithietic acid and biofilm formation to the probiotic activity of Phaeobacter inhibens
Source: BMC Microbiol. 2016 Jan 5;16:1. doi: 10.1186/s12866-015-0617-z (PMC4700733; doi:10.1186/s12866-015-0617-z)

**Additional File 1**. Electrospray ionization MS of purified TDA, structure shown on right, in the positive ion mode.  The spectrum shows expected losses of $H_2O$ and $CO_2H$.

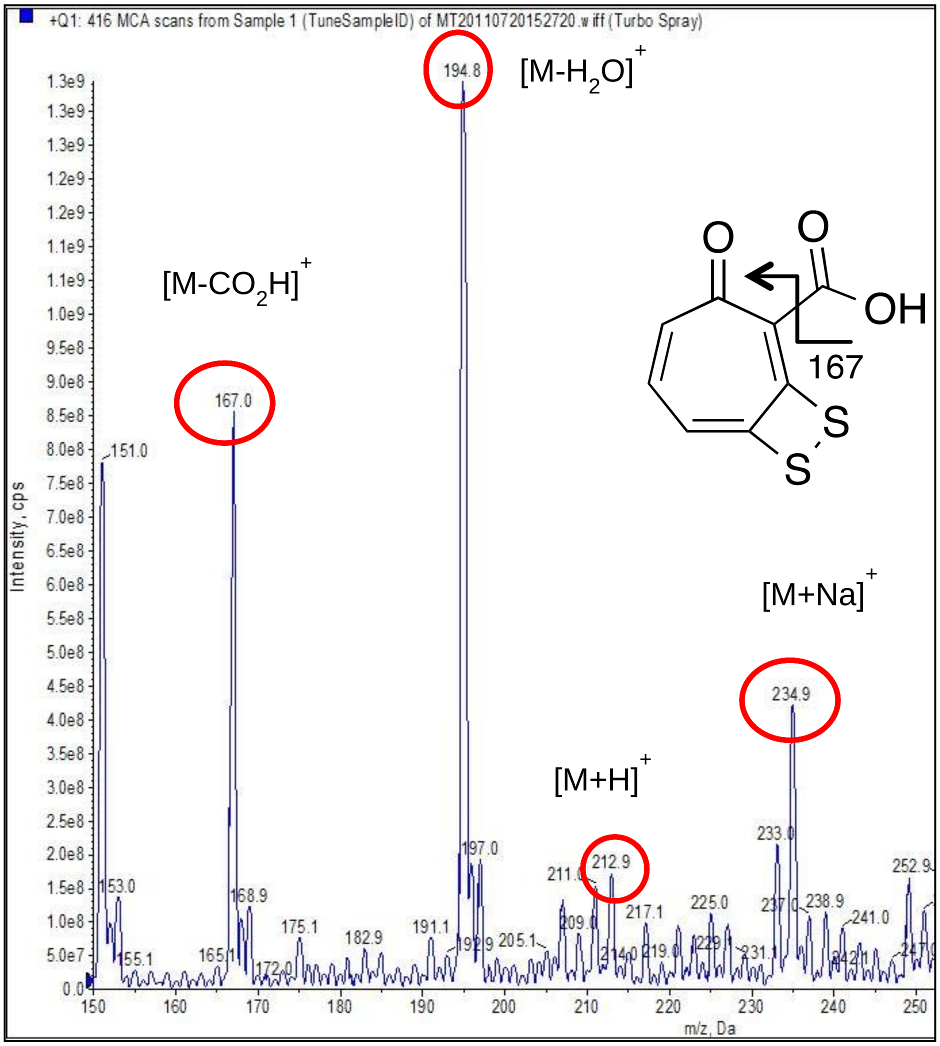

Supplement: Additional file 1: — A) Electrospray ionization MS of purified TDA, structure shown on right, in the positive ion mode. The spectrum shows expected losses of H2O and CO2H. (PDF 64 kb) [file 12866_2015_617_MOESM1_ESM.pdf]
