# Supplementary material for: Contributions of tropodithietic acid and biofilm formation to the probiotic activity of Phaeobacter inhibens
Source: BMC Microbiol. 2016 Jan 5;16:1. doi: 10.1186/s12866-015-0617-z (PMC4700733; doi:10.1186/s12866-015-0617-z)

Additional File 2. ^{1}H NMR spectrum (500 MHz, C6D6) for purified TDA. The spectrum is expanded to show the aromatic region of interest. Spectrum was referenced to residual benzene resonance at 7.16 ppm.

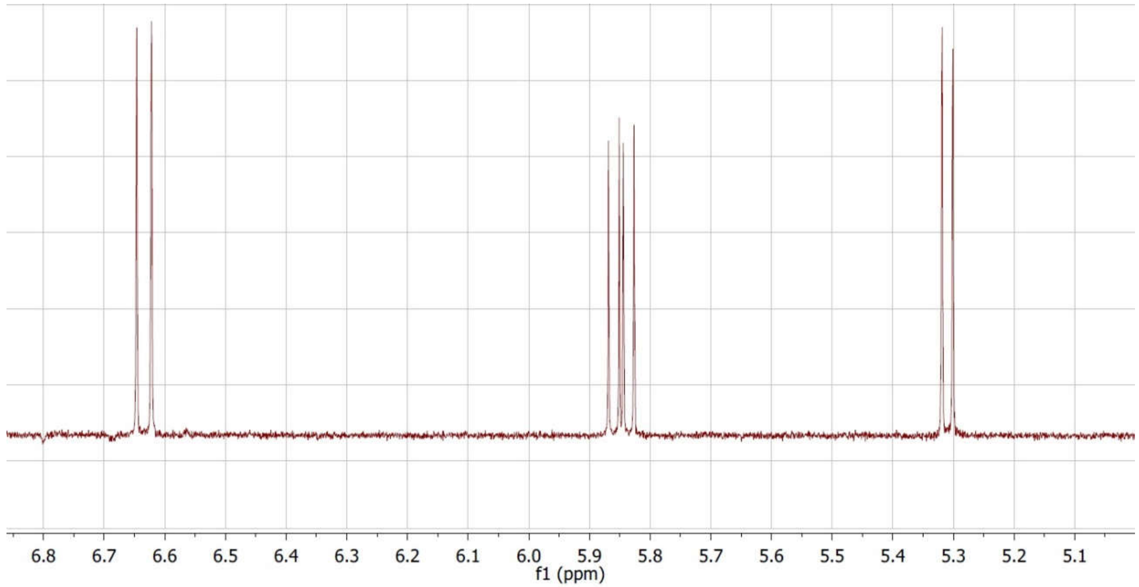

Supplement: Additional file 2: — 1H NMR spectrum (500 MHz, C6D6) for purified TDA. The spectrum is expanded to show the aromatic region of interest. Spectrum was referenced to residual benzene resonance at 7.16 ppm. (PDF 110 kb) [file 12866_2015_617_MOESM2_ESM.pdf]
