# Supplementary material for: Contributions of tropodithietic acid and biofilm formation to the probiotic activity of Phaeobacter inhibens
Source: BMC Microbiol. 2016 Jan 5;16:1. doi: 10.1186/s12866-015-0617-z (PMC4700733; doi:10.1186/s12866-015-0617-z)
Supplement: Additional file 3: — 1H NMR spectroscopic data of purified TDA from P. inhibens. Comparison is provided to data published by Liang (2003). (PDF 7 kb) [file 12866_2015_617_MOESM3_ESM.pdf]

Additional File 3. ^{1}H NMR spectroscopic data of purified TDA from *P. inhibens*. Comparison is provided to data published by Liang (2003).

H	*$ä_H$, m, J=Hz literature* (Liang 2003)	*$ä_H$ m, J=Hz experimental*
5	1H, 5.38, d, J=8.9	1H, 5.36, d J=9.0
6	1H, 5.90, dd, J=12.2, 8.9	1H, 5.89, dd, J=12.1, 8.8
7	1H, 6.63, d J=12.2	1H, 6.67, d, J=12.5
8	1H, 16.80 s	1H, 16.77 s
