# Supplementary material for: Contributions of tropodithietic acid and biofilm formation to the probiotic activity of Phaeobacter inhibens
Source: BMC Microbiol. 2016 Jan 5;16:1. doi: 10.1186/s12866-015-0617-z (PMC4700733; doi:10.1186/s12866-015-0617-z)

Additional File 4. A) Reversed-phase HPLC chromatograms of ethyl acetate extracts from *Phaeobacter* S4 mutant strains. B) Quantification of biofilm formation by measuring OD580 of crystal violet dye attached to the cells forming biofilms on glass tubes at 27°C under static condition. The data presented are average of two independent experiments and each independent experiment has three replicates.

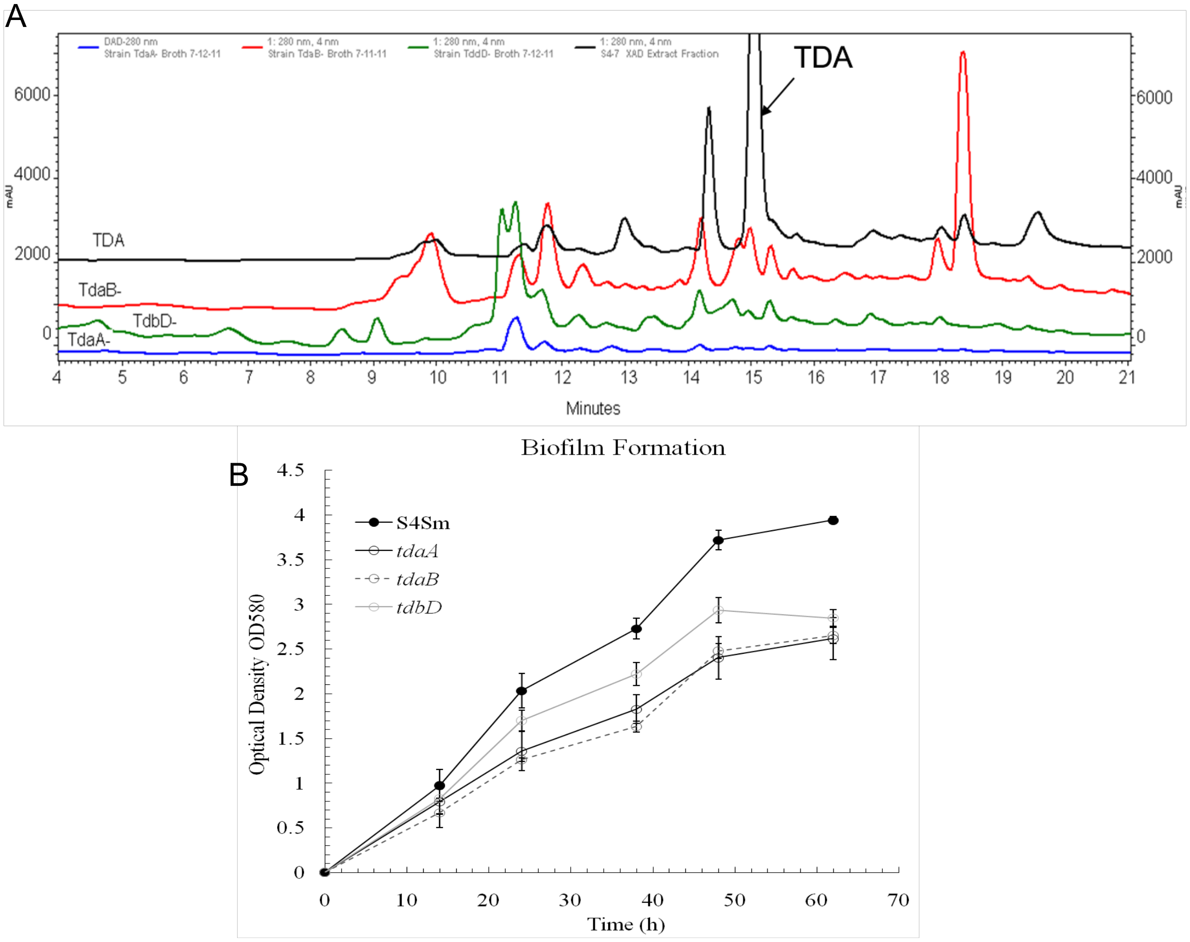

Supplement: Additional file 4: — A) Reversed-phase HPLC chromatograms of ethyl acetate extracts from Phaeobacter S4 mutant strains. B) Quantification of biofilm formation by measuring OD580 of crystal violet dye attached to the cells forming biofilms on glass tubes at 27 °C under static condition. The data presented are average of two independent experiments and each independent experiment has three replicates. Error bars represent one standard deviation. (PDF 83 kb) [file 12866_2015_617_MOESM4_ESM.pdf]
