# Supplementary material for: Contributions of tropodithietic acid and biofilm formation to the probiotic activity of Phaeobacter inhibens
Source: BMC Microbiol. 2016 Jan 5;16:1. doi: 10.1186/s12866-015-0617-z (PMC4700733; doi:10.1186/s12866-015-0617-z)

Additional File 5. Growth curve of Phaeobacter S4 strains under different conditions (static vs. shaking). Overnight culture of Phaeobacter cells were grown in YP30 media and then back-diluted into fresh YP30 1:1,000 dilution. Samples were taken at the indicated times and OD600 value were measured by a spectroscopy.

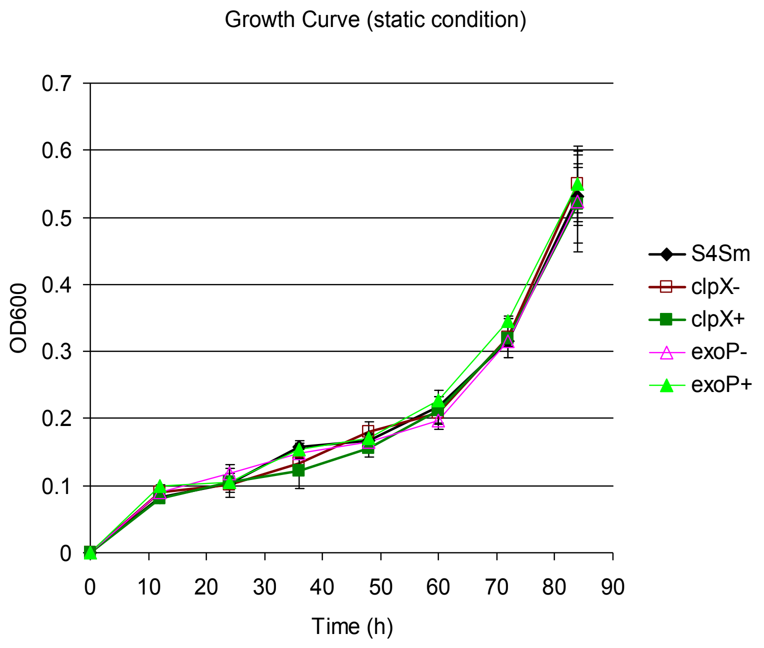

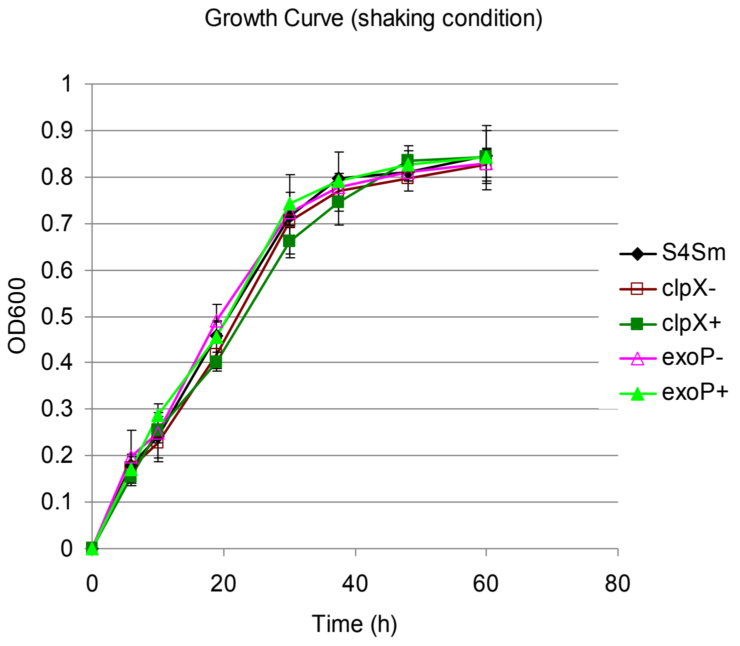

Supplement: Additional file 5: — Growth curve of Phaeobacter S4 strains under different conditions (static vs. shaking). Overnight culture of Phaeobacter cells were grown in YP30 media and then back-diluted into fresh YP30 1:1000 dilution. Samples were taken at the indicated times and OD600 value were measured by a spectroscopy. Error bars represent one standard deviation. (PDF 340 kb) [file 12866_2015_617_MOESM5_ESM.pdf]
