# Supplementary material for: Contributions of tropodithietic acid and biofilm formation to the probiotic activity of Phaeobacter inhibens
Source: BMC Microbiol. 2016 Jan 5;16:1. doi: 10.1186/s12866-015-0617-z (PMC4700733; doi:10.1186/s12866-015-0617-z)

Additional File 6. Effects of *V. anguillarum* NB10 on the growth of *P. gallaeciensis* strains in a competition assay without pre-colonization by *P. gallaeciensis*. The mixed cultures are: S4Sm-OFP with NB10Sm-GFP, *clpX*-OFP with NB10Sm-GFP and *exoP*-OFP with NB10Sm-GFP. Colonization and initial cell densities were as described in the Materials and Methods. A) Growth of sessile *P. gallaeciensis* cells (with NB10Sm) in a co-culture system and a monoculture control. B) Growth of planktonic *P. gallaeciensis* cells (with NB10Sm) in a co-culture system and a monoculture control. The data presented are average of two independent experiments and each independent experiment has three replicates.

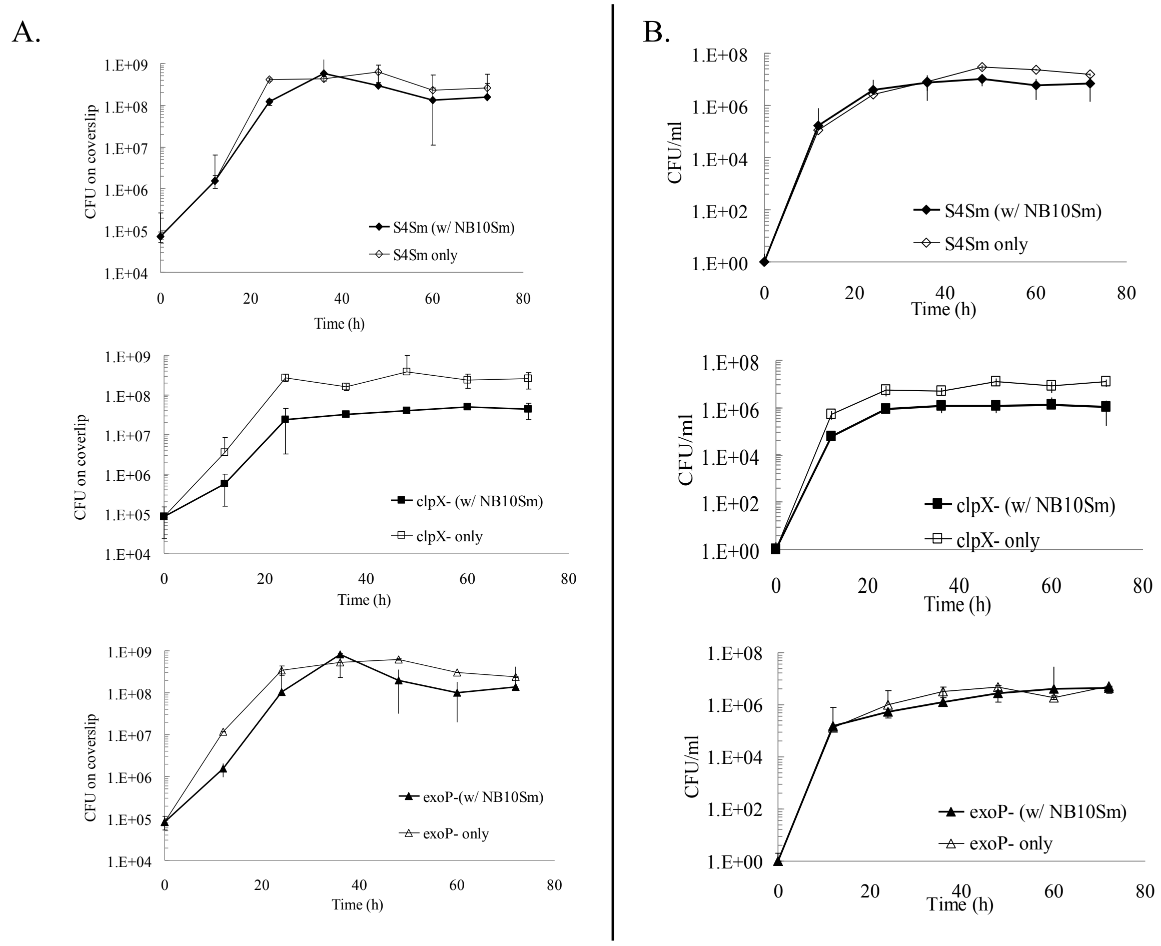

Supplement: Additional file 6: — Effects of V. anguillarum NB10 on the growth of P. inhibens strains in a competition assay without pre-colonization by P. inhibens . The mixed cultures are: S4Sm-OFP with NB10Sm-GFP, clpX-OFP with NB10Sm-GFP and exoP-OFP with NB10Sm-GFP. Colonization and initial cell densities were as described in the Materials and Methods. A) Growth of sessile P. inhibens cells (with NB10Sm) in a co-culture system and a monoculture control. B) Growth of planktonic P. inhibens cells (with NB10Sm) in a co-culture system and a monoculture control. The data presented are average of two independent experiments and each independent experiment has three replicates. Error bars represent one standard deviation. (PDF 362 kb) [file 12866_2015_617_MOESM6_ESM.pdf]
