# Supplementary material for: Contributions of tropodithietic acid and biofilm formation to the probiotic activity of Phaeobacter inhibens
Source: BMC Microbiol. 2016 Jan 5;16:1. doi: 10.1186/s12866-015-0617-z (PMC4700733; doi:10.1186/s12866-015-0617-z)

Additional File 7. Competition assay between *Phaeobacter* S4Sm strains and *V. anguillarum* NB10Sm without pre-colonization by *Phaeobacter*. The mixed cultures are S4Sm-OFP with NB10Sm-GFP, *clpX*-OFP with NB10Sm-GFP and *exoP*-OFP with NB10Sm-GFP. A) Single channel and merged confocal microscopy images of mixed biofilm development by OFP-producing strains (S4Sm, *clpX* mutant or *exoP* mutant) and GFP-producing *V. anguillarum* NB10Sm-GFP on the surface of glass coverslip at 48 h. The data presented is from a representative experiment of two independent experiments. B) Growth of sessile NB10Sm in co-culture system with different *Phaeobacter* strains. C) Comparison of growth of sessile *V. anguillarum* (NB10Sm) in different co-culture system and monoculture control. D) Growth of planktonic *V. anguillarum* (NB10Sm) in co-culture system with different *Phaeobacter* strains. E) Comparison of growth of planktonic *V. anguillarum* (NB10Sm) in different co-culture system and monoculture control. The data presented are average of two independent experiments and each independent experiment has three replicates.

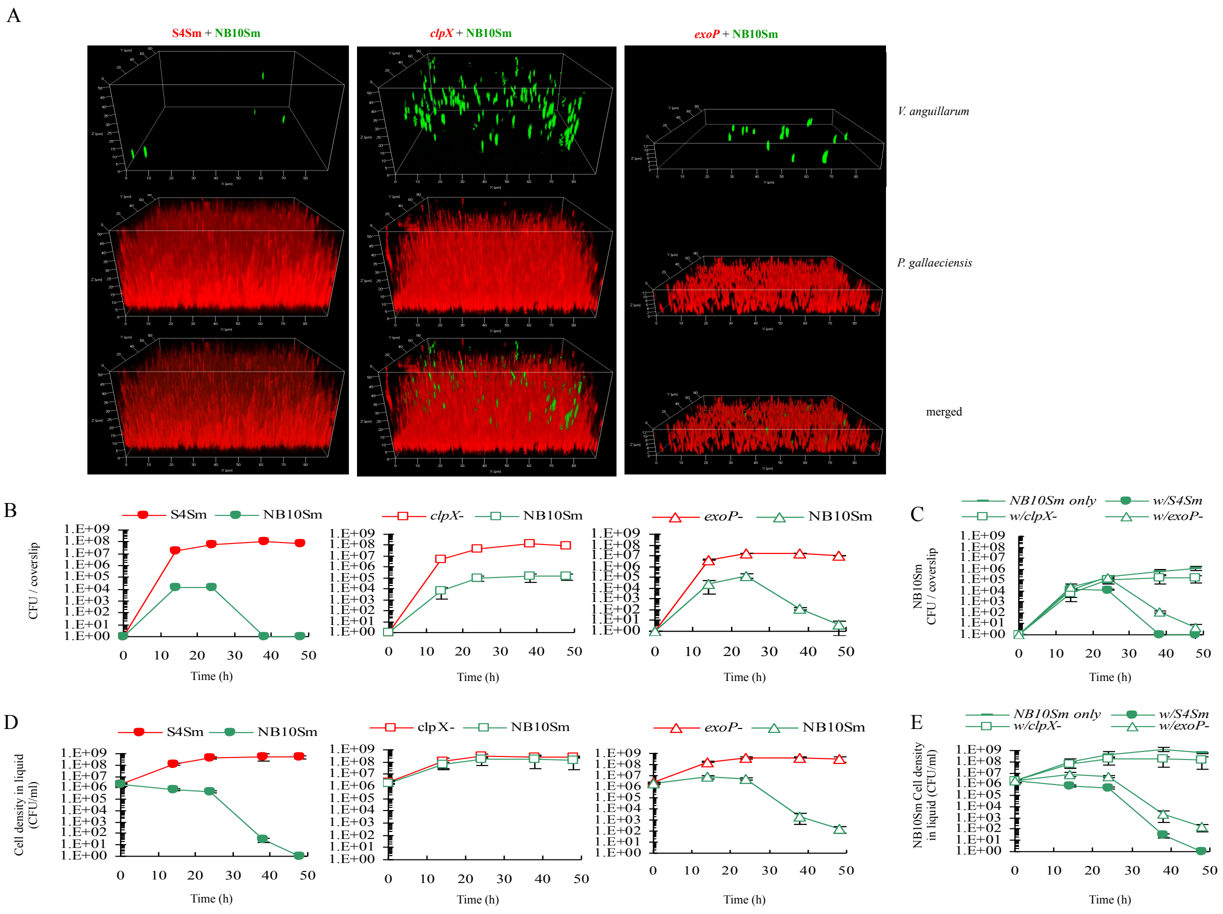

Supplement: Additional file 7: — Competition assay between P. inhibens S4Sm strains and V. anguillarum NB10Sm without pre-colonization by P. inhibens . The mixed cultures are S4Sm-OFP with NB10Sm-GFP, clpX-OFP with NB10Sm-GFP and exoP-OFP with NB10Sm-GFP. A) Single channel and merged confocal microscopy images of mixed biofilm development by OFP-producing strains (S4Sm, clpX mutant or exoP mutant) and GFP-producing V. anguillarum NB10Sm-GFP on the surface of glass coverslip at 48 h. The data presented is from a representative experiment of two independent experiments. B) Growth of sessile NB10Sm in co-culture system with different Phaeobacter strains. C) Comparison of growth of sessile V. anguillarum (NB10Sm) in different co-culture system and monoculture control. D) Growth of planktonic V. anguillarum (NB10Sm) in co-culture system with different Phaeobacter strains. E) Comparison of growth of planktonic V. anguillarum (NB10Sm) in different co-culture system and monoculture control. The data presented are average of two independent experiments and each independent experiment has three replicates. Error bars represent one standard deviation. (PDF 1811 kb) [file 12866_2015_617_MOESM7_ESM.pdf]
